# Free radical inhibition and total phenolic content in *Fomitopsis betulina* mycelium extract under different cultivation conditions

**DOI:** 10.3389/ffunb.2026.1735209

**Published:** 2026-01-23

**Authors:** Tetiana Zaichenko, Victor Barshteyn, Mustafa Sevindik, Tetiana Krupodorova

**Affiliations:** 1 Department of Plant Food Products and Biofortification, Institute of Food Biotechnology and Genomics of the National Academy of Sciences of Ukraine, Kyiv, Ukraine; 2 Department of Biology, Faculty of Engineering and Natural Sciences, University of Osmaniye Korkut Ata, Osmaniye, Türkiye

**Keywords:** birch polypore, DPPH, growth *in vitro*, mycelial extract, phenols

## Abstract

**Introduction:**

Given the growing recognition of *Fomitopsis betulina* for its bioactive potential, the influence of cultivation parameters on its mycelial development, metabolite production in submerged culture, and associated antioxidant activity remains insufficiently explored.

**Methods:**

This study investigated the effects of various cultivation parameters on biomass accumulation, total phenolic content (TPC), and free radical scavenging activity, assessed using the Folin–Ciocalteu and DPPH assays respectively.

**Results and Discussion:**

Among solvents tested, methanol and 70% ethanol were most effective for phenolic extraction, yielding 20.54±0.11 and 19.39±0.14mg GAE/g, respectively, while some solvents demonstrated strong DPPH inhibition (≥90%). A cultivation at 25°C supported optimal biomass accumulation (5.23±0.10g/L), phenolic compound total yield (101.10mg GAE/L), and antioxidant activity (91.66±0.40%). Static cultivation conditions promoted surface mycelial growth and resulted in the highest biomass yield (5.28±0.15g/L), strong DPPH inhibition (≥90%), and phenolic synthesis (101.75mg GAE/L). Among carbon sources, maltose favored biomass formation, whereas xylose led to the highest DPPH inhibition (89.68±0.91%) and TPC (16.08±0.06mg GAE/g; total yield: 15.92mg GAE/L). Of the nitrogen sources evaluated, ammonium sulfate supported the greatest biomass accumulation (2.64±0.21g/L), while ammonium nitrate enhanced antioxidant activity (80.54±3.10%). Although urea produced the highest TPC per gram of dry biomass (11.32±0.05mg GAE/g), ammonium sulfate resulted in the highest phenolic total yield (18.43mg GAE/L). An initial medium pH of 6.0 was identified as optimal for maximizing biomass growth, phenolic compound production, and antioxidant capacity. The cultivation parameters were ranked in order of influence as: temperature > duration of static cultivation > pH > duration of agitation > carbon source > nitrogen source. These findings provide a foundation for the targeted optimization of cultivation conditions to enhance biomass production, phenolic compound accumulation, and antioxidant activity in *F. betulina* (GenBank accession: PQ184655). The results contribute to the broader understanding of fungal secondary metabolite production and support future applications in biotechnology and functional food development. .

## 1 Introduction

Medicinal mushrooms of the genus *Fomitopsis* contain numerous biologically active compounds, which offer significant pharmacological potential, including antimicrobial, anti-inflammatory, antioxidant, and immunomodulatory activities, and are used in the treatment of cancer and cardiovascular diseases, as well as modulation of the immune system (Karunarathna et al., 2025). Additionally, *Fomitopsis* species such as *F. pinicola* and *F. betulina*, contribute to biotechnological advancements, playing a role in the development of pharmaceuticals and functional foods due to the unique properties of their metabolites (Krupodorova et al., 2024a; Kizitska et al., 2024).

Among *Fomitopsis* species, the Birch polypore (*Fomitopsis betulina* (Bull.) B.K. Cui, M.L. Han, and Y.C. Dai) is a well-known medicinal mushroom used for centuries in Europe and Asia to treat infectious diseases, inflammatory processes and gastrointestinal disorders. Recent research has confirmed the mushroom’s significant phytochemical composition and pharmacological potential (Li et al., 2024). Fruiting bodies and mycelium contain various biologically active substances, which contribute to its antimicrobial, anti-inflammatory, antioxidant, and anticancer activities (Tiupova et al., 2025). Mechanisms such as immunomodulation, suppression of inflammatory signaling, inhibition of microbial proliferation, and induction of apoptosis in tumor cells underlie the above-described activities. The use of *F. betulina*-based ingredients in the creation of functional foods and dietary supplements is in line with one of the important modern trends in healthcare, healthy eating (Yang et al., 2025). The numerous therapeutic properties of *F. betulina* suggest a potential role for this mushroom in the prevention and treatment of chronic and degenerative diseases, particularly those associated with oxidative stress and inflammation.

Antioxidants supplied to the human body from external sources help the endogenous antioxidant system neutralize excess reactive oxygen species (ROS), preventing cell damage and oxidative stress (Liu et al., 2018). In this aspect, mushroom raw materials are one of the available sources of natural antioxidants such as phenols, flavonoids, polysaccharides, peptides, sterols, pigments, and alkaloids (Arslan et al., 2023). It should be noted that some exogenous antioxidants such as amino acid ergothioneine and the compound glutathione are found only in mushrooms. The antioxidant activity of mushrooms can be demonstrated *in vitro* using DPPH (2,2-diphenyl-1-picrylhydrazyl), ABTS (2,2’-azino-bis(3-ethylbenzothiazoline-6-sulfonic acid)), nitric oxide, hydroxyl, superoxide, and hydrogen peroxide assays. Notably, in *in vivo* models, the use of mushroom antioxidants increases the levels of various antioxidant enzymes, such as catalase, glutathione peroxidase, and superoxide dismutase, and reducing malondialdehyde levels. Thus, mushroom-derived antioxidants have potential applications in the food, cosmetic, and pharmaceutical industries (Yang et al., 2025).

Previous studies have determined fatty acids, indole compounds, sterols, triterpenes, and phenolic acids in fruiting bogy and mycelial extracts of *F. betulina*, mainly focusing on anti-inflammatory as well as cytotoxic activity (Sułkowska-Ziaja et al., 2018). The differences in antioxidant activity and total phenolic content among various strains of *F. betulina* highlight the strain-specific nature of the fungi in synthetizing certain biologically active compounds and enhancing their therapeutic activities (Zaichenko et al., 2025). The importance of identifying promising producing strains cannot be overstated, as it represents the first and the most crucial step toward downstream applications. Along with this, the proven possibility of adjusting the controlled abiotic cultivation to increase the yield of the target metabolite and enhance its antioxidant activity (Shu and Lung, 2008; Vamanu, 2013; Dang et al., 2018; Krupodorova et al., 2024b; Elesseily et al., 2025) makes it promising to obtain fungal mycelial mass rather than growing fruiting bodies. These interrelated key points led to the aim of our work: to evaluate the influence of cultivation condition on biomass and phenols production, as well as enhancement of free radical scavenging activity of *F. betulina*.

## 2 Materials and methods

### 2.1 Sample collection and fungal isolation

The fruiting body of *Fomitopsis betulina* was collected in the Holosiivskyi National Nature Park Kyiv, Ukraine. The fungal sample was transported to the laboratory and immediately treated with sterile distilled water and a 70% ethyl alcohol solution for the culture isolation. Under aseptic conditions, small pieces were excised from the interior of the fruiting body using tweezers sterilized by flame, and transferred to the surface of the Malt Extract Agar (MEA, Thermo Fisher Scientific, USA) medium. The obtained pure culture was subsequently deposited in the IBK Mushroom Culture Collection of the M.G. Kholodny Institute of Botany of the National Academy of Sciences of Ukraine under strain number 2777 (Bisko et al., 2024). The fungi genetic sequences of identification have been deposited in GenBank under the accession number PQ184655 (data submitted before this publication). The culture was stored on MEA slant at 4°C for future use.

### 2.2 Preparation of inoculum

The inoculum of *F. betulina* was transferred from a storage slant to a Petri dish containing Glucose Peptone Yeast Agar (GPYA) composed of the following (g/L): 25.0 glucose, 3.0 peptone, 2.0 yeast extract, 1.0 K_2_HPO_4_, 1.0 KH_2_PO_4_, 0.25 MgSO_4_·7H_2_O, and 10.0 agar. For cultivation under static and submerged shaking conditions, Glucose Peptone Yeast Broth (GPYB), was used.

For static cultivation, 250 mL Erlenmeyer flasks containing 50 mL of GPYB medium were autoclaved for 15 minutes at 121 °C. Each flask was then inoculated with three mycelial discs (8 mm in diameter) that were aseptically cut out from a 10-day-old *F. betulina* culture grown on a Petri dish using a sterile punch.

For submerged cultivation with shaking, mycelium from a 10-day-old *F. betulina* culture on GPYA was collected and ground in a sterile environment using a homogenizer (MPW-120, Mechanika Precyzyjna, Warszawa, Poland) with 250 ml of GPYB. The resulting suspension was then divided into 5 mL aliquots and transferred to each sterile Erlenmeyer flask containing 50 ml of GPYB. The flasks were placed on an orbital shaker at 120 rpm, in the dark, at 25 °C from 3 to 15 days at 2-day intervals.

### 2.3 Cultivation conditions

To evaluate the impact of temperature, the flasks with GPYB were inoculated with three mycelial discs and incubated under static conditions for 14 days at different temperatures (20°C, 25°C, and 30°C) in incubators.

To assess the cultivation time and method, another flask containing the GPYB medium were inoculated with the prepared inoculum (three mycelial disks or 5 ml of suspension) and incubated at 25°C. The incubation time was determined by monitoring of the fungal growth dynamics (for static cultivation, incubation was carried out from 7 to 35 days at 7-day intervals, while for submerged shaking cultivation, it was carried out from 3 to 15 days at 2-day intervals).

To determine the effect of pH level, other flasks with the GPYB were inoculated with three mycelial discs and cultivated under static conditions at 25°C for 14 days at different pH levels (ranging from 2.5 to 8.0, with 0.5 intervals). The pH of the GPYB medium was adjusted using 1M HCl and 1M NaOH and was monitored before and after autoclaving using a digital pH meter.

To asses the impact different nutritional sources was prepared a base medium consisting of (g/L): 10.0 glucose, 0.4 asparagine, 1.0 KH_2_PO_4_, and 0.5 MgSO_4_×7 H_2_O. Glucose in the base medium was substituted with equivalent carbon content from various carbon sources (dextrose, fructose, galactose, mannitol, arabinose, xylose, sucrose, lactose, maltose, soluble starch, or cellulose). Similarly, asparagine was replaced with an equivalent amount of the respective nitrogen source (peptone, yeast extract, urea, ammonium nitrate, ammonium sulfate, potassium nitrate, sodium nitrate, or sodium nitrite). The pure carbon or nitrogen content per liter of medium was calculated based on the molecular weight of the compounds and the percentage of carbon or nitrogen in the compound, which were calculated for every substance according to following equation:


$$\%(X)=\;\frac{A_{r}(X)\times n(X)}{M_{r}};$$

where *%(X)* – the percentage of investigated element (carbon or nitrogen) in the compound;

*A_r_(X)* – atomic mass of investigated element;

*n(X)* – amount of investigated element atoms in certain compound;

*M_r_* – the molecular mass of certain compound.

The mass of the substitute compound in the nutritional medium was determined as follows:


$$m(X)=\frac{\%(B)\times m(B)}{\%(X)};$$

where *m(X)* – the mass of the substitute compound needed;

*%(B)* – the percentage of elements in the base medium (carbon in glucose or nitrogen in asparagine, respectively);

*m(B)* – the mass of the components in the base medium (carbon or asparagine, respectively);

*%(X)* – the percentage of investigated element (carbon or nitrogen) in the substitute compound.

Flasks with the prepared medium were inoculated with three mycelial discs and incubated under static conditions at 25 °C for 28 days.

### 2.4 Biomass amount determination

After each cultivation period, the mycelium was harvested from the culture medium filtration using Whatman filter paper No. 4. The filtered mycelium was subsequently washed with distilled water and then dried to a constant weight at 85°C. Fungal growth was quantified as the mycelial biomass (g/L) in terms of absolute dry weight (a.d.w.).

### 2.5 Crude extract production

The mycelium for analysis was obtained by cultivating *F. betulina* in GPYB medium (pH 6.0) under static conditions at 25°C for 14 days. After cultivation, the mycelium was separated from the culture medium by filtration using Whatman filter paper No. 4, washed with distilled water, and dried at 60°C to constant weight. The dried mycelium was then powdered in a laboratory ball mill to a particle size of approximately 50-100 µm. To compare solvent-dependent extraction efficiency and identify the most suitable solvent, the powder was extracted at a 1:10 (w/v) ratio using chloroform, ethyl acetate, 96% ethanol, 70% ethanol, methanol, and distilled water. The mixtures were shaken for 48 hours at 100 rpm and 30°C. The supernatants were collected after centrifugation at 4000 rpm for 10 minutes, followed by filtration through a 25 μm pore size filter (class 4 filter paper). The resulting extracts were stored at 4°C for up to one week prior to the determination of antioxidant activity (AOA) and total phenolic content (TPC). Following the selection of 70% ethanol as the optimal solvent, it was used to prepare the extracts necessary for assessing the impact of *F. betulina* cultivation conditions.

### 2.6 Antioxidant activity determination by DPPH·assay

AOA was assessed using method involving 2,2-diphenyl-1-picrylhydrazyl (DPPH, Thermo Fisher Scientific, USA) (Blois, 1958). The reduction of the DPPH radical was quantified at 517 nm using a UV-1800 PC spectrophotometer (Shanghai, China), in accordance with the procedure described in our previous study (Krupodorova et al., 2024b). Ascorbic acid (0.3 mg/mL) served as the positive control, while the negative control was prepared by substituting the sample with an equivalent volume of methanol in the reaction mixture. The results were expressed as the percentage inhibition of DPPH radical scavenging, calculated using the absorbance values in the following formula:


$$DPPH\;radical\;\;scavennging\;\;activity(\%)=[\frac{Асontrol-Аsample}{\text{A}control}]\times 100$$

### 2.7 Total phenolic content determination

TPC was estimated as reducing capacity using the Folin–Ciocalteu assay and expressed as gallic acid equivalents (GAE) per gram of dry mycelium (Folin and Ciocalteu, 1927; Singleton and Rossi, 1965). The detailed procedure for determination is described in our previous study (Zaichenko et al., 2025). After incubation 60 minutes, the absorbance was measured at 750 nm using a UV-1800 PC spectrophotometer (Shanghai, China). The concentration of phenolic compounds was calculated based on a calibration curve (y = 0.0709x + 0.0498; R^2^ = 0.9783) constructed using triplicate measurements of gallic acid as the standard and positive control. Gallic acid concentrations ranging from 2 to 40 μg/ml were used. All reagents (Folin-Ciocalteu phenol reagent, sodium carbonate, and distilled water), excluding the extract, served as the negative control. Additionally, the total yield of phenolic compounds after cultivation was calculated and expressed as milligrams of GAE per liter of culture medium.

### 2.8 Statistical analysis

All measurements were performed from three biological replicates, and the results are expressed as the mean±standard deviation (SD). Statistical significance was determined using ANOVA and at a p-value ≤ 0.05. The Pearson correlation coefficients (r) for biomass, AOA, and TPC under different cultivation conditions of *F. betulina* were calculated using the online statistical software Statistics Kingdom, available at https://www.statskingdom.com/.

## 3 Results

### 3.1 Isolation and culture characterization of *F. betulina*

**Figure 1:**
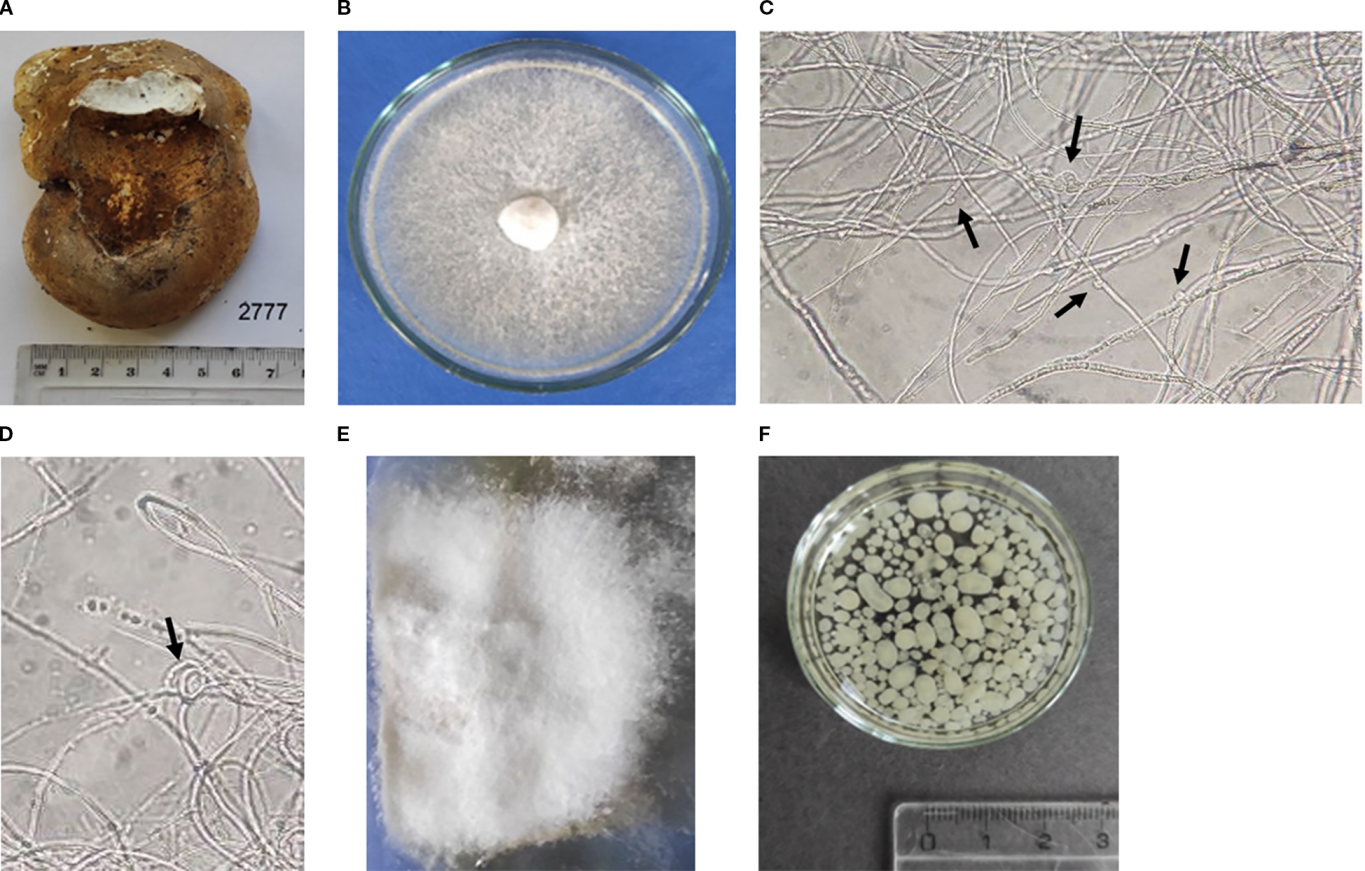
*Fomitopsis betulina* fruiting body **(A)**, isolated *F*. *betulina* colony on MEA medium **(B)**, microscopy of mycelium: clamp connections **(C)**, hyphal loops **(D)** at ×400 magnification, mycelium under static cultivation during 14 days **(E)** and submerged shaking cultivation during 11 days **(F)**.

Fungal isolate was readily obtained in pure culture from collected fruiting bogy (**Figure 1A**) using standard method on agar plate. Macroscopic characteristics were examined on Malt Extract Agar (MEA) at 25 °C after 9 days of incubation. The isolate formed circular, white, floccose colonies without exudate production and a mildly astringent odor. The aerial mycelium of medium density was evenly distributed and intertwined. (**Figure 1B**). The colony margin was entire and appressed. Under light microscopic examination, the isolate showed branched vegetative hyphae with both large and small clamp connections (**Figure 1C**) and hyphal loops (**Figure 1D**). The morphology of *F. betulina* mycelium was typical of basidiomycetes under static conditions, mycelial growth occurred fully or partially at the liquid surface (**Figure 1E**), whereas under dynamic cultivation (shaking), the mycelium was fully submerged and formed pellets (**Figure 1F**).

### 3.2 Selection of extractant

To obtain *F. betulina* mycelial extract with the highest concentration of bioactive compounds, different solvents (distilled water, 96% and 70% ethanol, methanol, chloroform and ethyl acetate) were tested. The result of AOA, TPC, and the total yield of phenolic compounds of the obtained extracts were considered to verify the effectiveness of the extraction process. The highest DPPH free radical’s inhibition were obtained using methanol (91.87±0.67%), water (91.01±0.38%) and 70% ethanol (90.06±0.90%) (**Table 1**).

**Table 1:** The effect of extractant on AOA and phenols level.

Solvent	AOA, %	TPC, mg GAE/g	Total phenolic yield, mg GAE/L
Chloroform	19.14±1.20	0.44±0.02	2.29±0.10
Ethyl acetate	79.22±1.80	3.68±0.03	19.14±0.16
Ethanol 96%	75.64±2.30	10.85±0.30	56.42±1.56
Ethanol 70%	90.06±0.90	19.39±0.14	100.83±0.73
Methanol	91.87±0.67	20.54±0.11	106.81±0.57
Distilled water	91.01±0.38	8.01±0.05	41.65±0.26

AOA, antioxidant activity, TPC, total phenolic content (per gram of dry biomass), GAE, gallic acid equivalent.

However, the most effective extraction of phenolic compounds was achieved using methanol (20.54±0.11 mg GAE/g dry mycelium with a total yield of 106.81±0.57 mg GAE/L) and 70% ethanol (19.39±0.14 mg GAE/g with a total yield of 100.83±0.73 mg GAE/L). Considering the extraction performance together with solvent polarity, toxicity, and ease of handling, 70% ethanol was selected for subsequent experiments.

### 3.3 Effect of temperature

**Figure 2:**
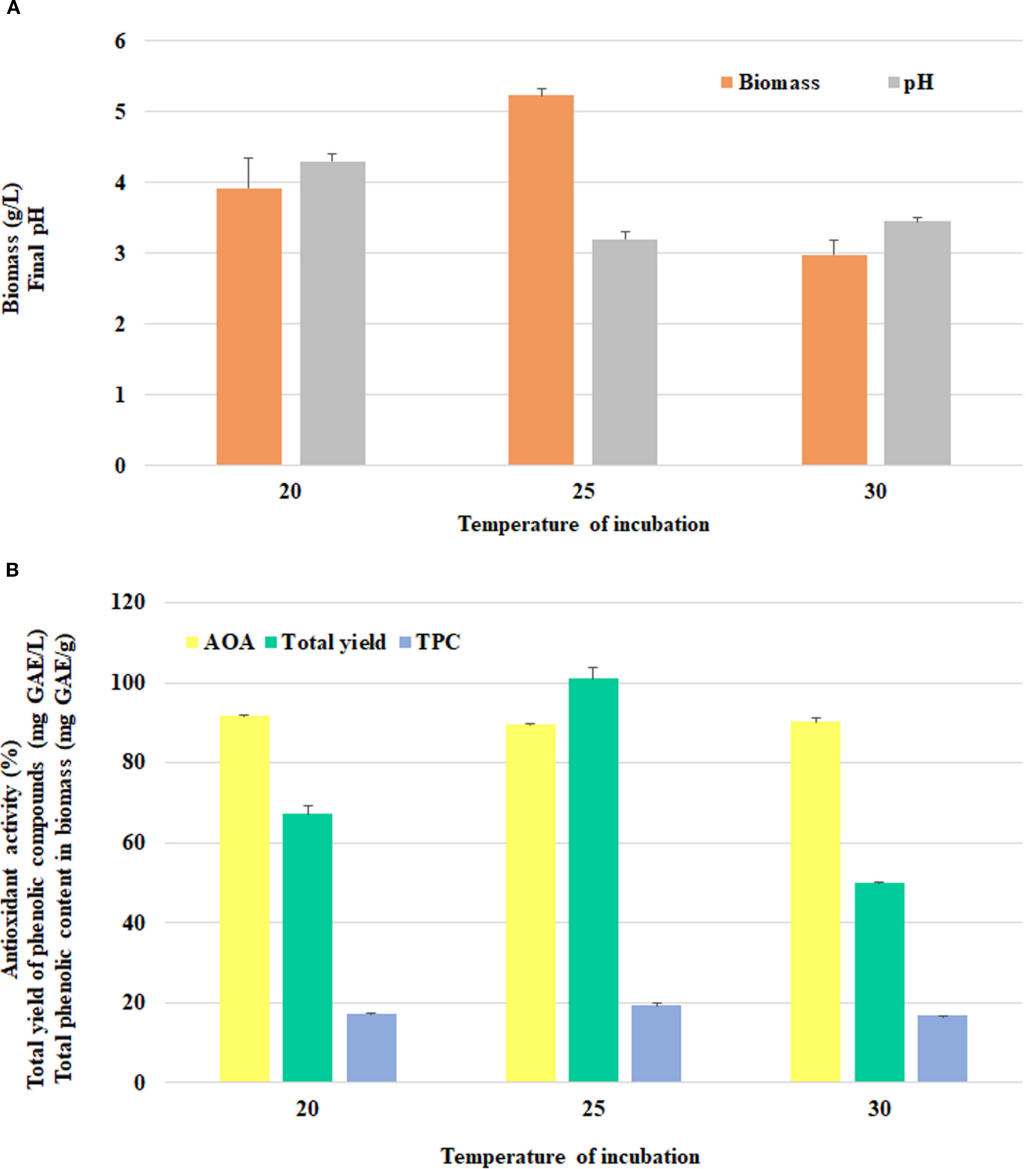
The effect of incubation temperature on *F*. *betulina*: **(A)** biomass growth and final pH of cultural media; **(B)** phenols accumulation level and AOA.

As fungal growth and metabolic activity were significantly dependent on environmental temperature, the optimal cultivation temperature was subsequently evaluated. *F. betulina* exhibited the highest biomass accumulation (5.23±0.10 g/L) at 25°C (**Figure 2A**). In all treatments, the final pH of the culture medium remained acidic.

Free radical inhibition levels as well as TPC of mycelial extracts varied slightly across temperatures (**Figure 2B**). The effect of temperature on total phenolic yield was strongest, with the highest total phenolic yield achieved at 25°C (101.10±2.60 mg GAE/L).

### 3.4 Effect of cultivation method over time

**Figure 3:**
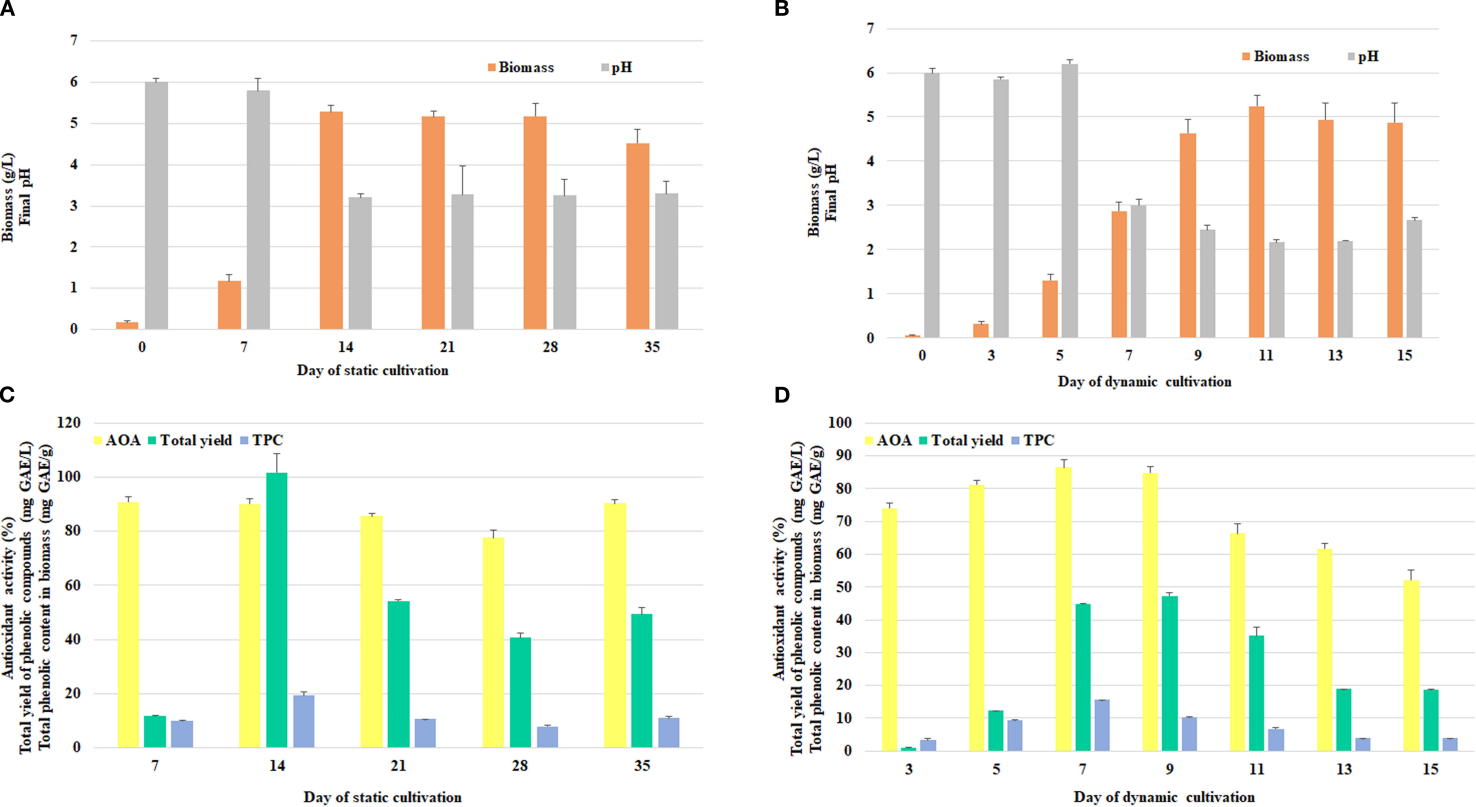
The effect of cultivation method and duration on *F*. *betulina* biomass growth and final pH of cultural media under static **(A)** and dynamic shaking **(B)** conditions, and on duration on *F*. *betulina* AOA and phenols accumulation level under static **(C)** and dynamic shaking **(D)** conditions.

In the next stage of the study, the submerged liquid cultivation of *F. betulina* was investigated under different conditions. These conditions included static cultivation, with either fully or partially surface-grown mycelium, and dynamic cultivation with shaking, which results in fully submerged mycelium. Notably, both cultivation methods resulted in a quantitatively similar maximum amount of mycelium obtained: 5.28±0.15 g/L after 14 days of static incubation (**Figure 3A**) and 5.25±0.24 g/L after 11 days of dynamic incubation (**Figure 3B**).

However, the changes in pH during cultivation were distinct between the two cultivation approaches. While static cultivation led to a change in pH from 6.0 to 3.2–3.3 after 14 days, dynamic cultivation caused a more significant drop in pH to 2.2–3.0 by day 7.

During the cultivation period, extracts from mycelium grown under static conditions consistently exhibited relatively high antioxidant activity (AOA), maintaining levels above 70% (**Figure 3C**). This stable AOA suggests that static cultivation conditions may support the sustained production of bioactive compounds, which could be beneficial for further applications.

The best results (≥90% inhibition) were obtained from the mycelium on days 7, 14 and 35. The maximum accumulation of phenols was achieved on day 14 (19.27±1.31 mg GAE/g, total 101.75 mg GAE/L). A decline in the assessed parameters was observed on days 21 and 28 of cultivation. However, rising levels of free radical inhibition and phenolic content were detected again on day 35.

The optimal cultivation period for the assessed parameters in the extracts under constant shaking conditions was established to be days 7 and 9 (**Figure 3D**). On these period, the AOA was 86.53±2.20% and 84.89±1.90%, respectively, and the maximum phenols yield was 15.60±0.03 mg GAE/g and 44.93±0.09 mg GAE/L (day 7), and 10.19±0.22 mg GAE/g and 47.28±1.01 mg GAE/L (day 9). These results were statistically significant, thereby supporting the conclusion that days 7 and 9 represent the optimal cultivation time points.

### 3.5 Effect of carbon and nitrogen sources

**Figure 4:**
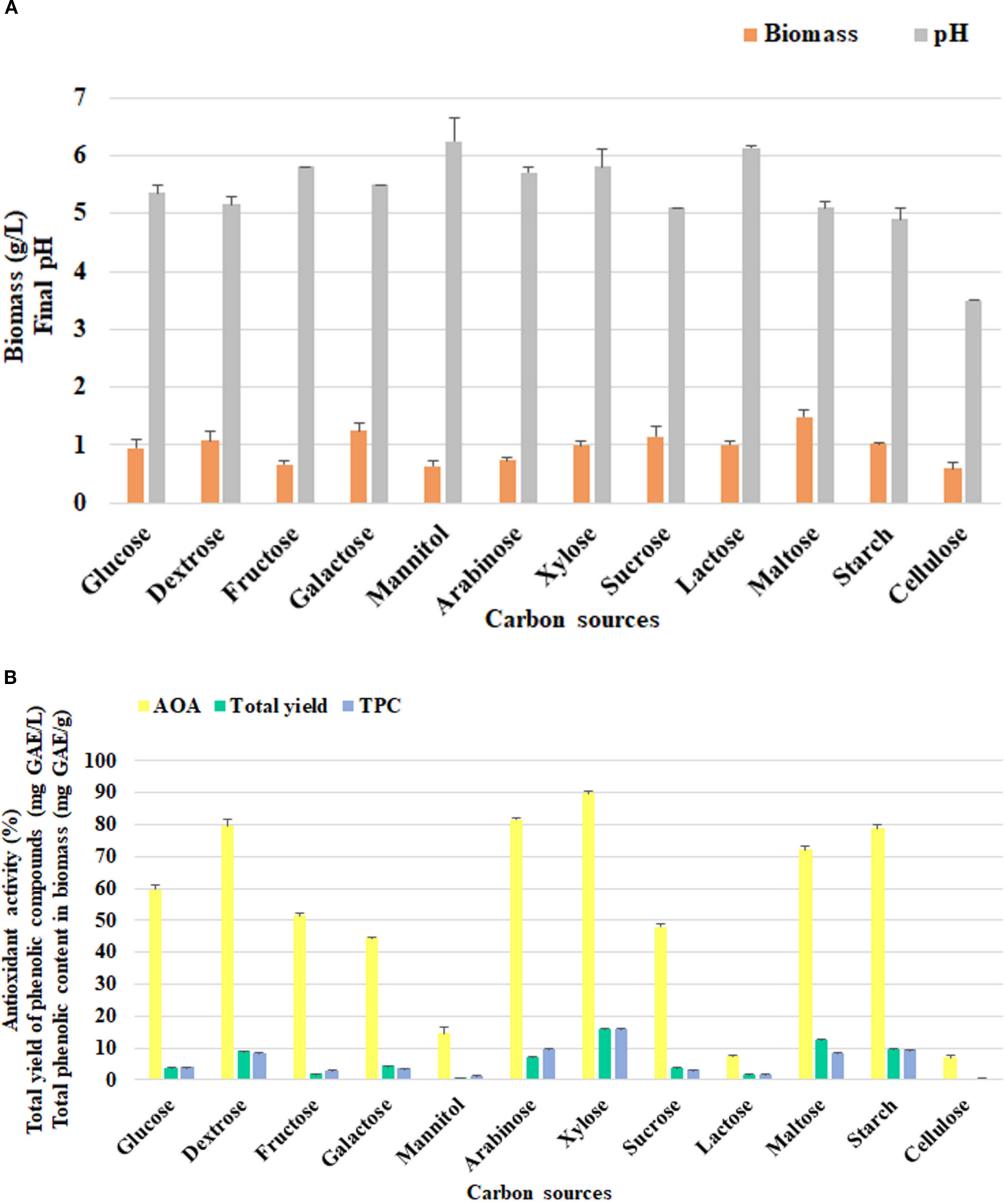
The effect of carbon nutritional sources on *F*. *betulina*: **(A)** biomass growth and final pH of cultural media; **(B)** phenols accumulation level and AOA.

To identify the optimal nutritional sources for the growth and development of *F. betulina*, twelve different carbon sources were tested, including hexoses, pentoses, sugar alcohol, disaccharides and polysaccharides. The biomass level varied depending on the carbon source used (**Figure 4A**). Maltose was identified as the most favorable carbon source for biomass formation, yielding 1.49±0.11 g/L. Biomass production on galactose and sucrose was slightly lower, though not significantly different between the two. The fungus exhibited the least growth on fructose, mannitol, arabinose, and cellulose.

The final pH after cultivation showed a range from slightly acidic to moderately acidic across most conditions. However, when the cultivation was carried out with cellulose, the pH value dropped significantly, reaching a strongly acidic level of 3.5. This variation suggests that the presence of cellulose may have a pronounced effect on the metabolic processes of the culture, leading to a more acidic environment.

In contrast, the optimal carbon sources for the assessed parameters were different (**Figure 4B**). The maximum results were achieved with xylose (AOA 89.68±0.91%, TPC 16.08±0.06 mg GAE/g and total phenolic yield 15.92±0.06 mg GAE/L). High levels of antioxidant activity were also obtained using arabinose, dextrose and starch.

**Figure 5:**
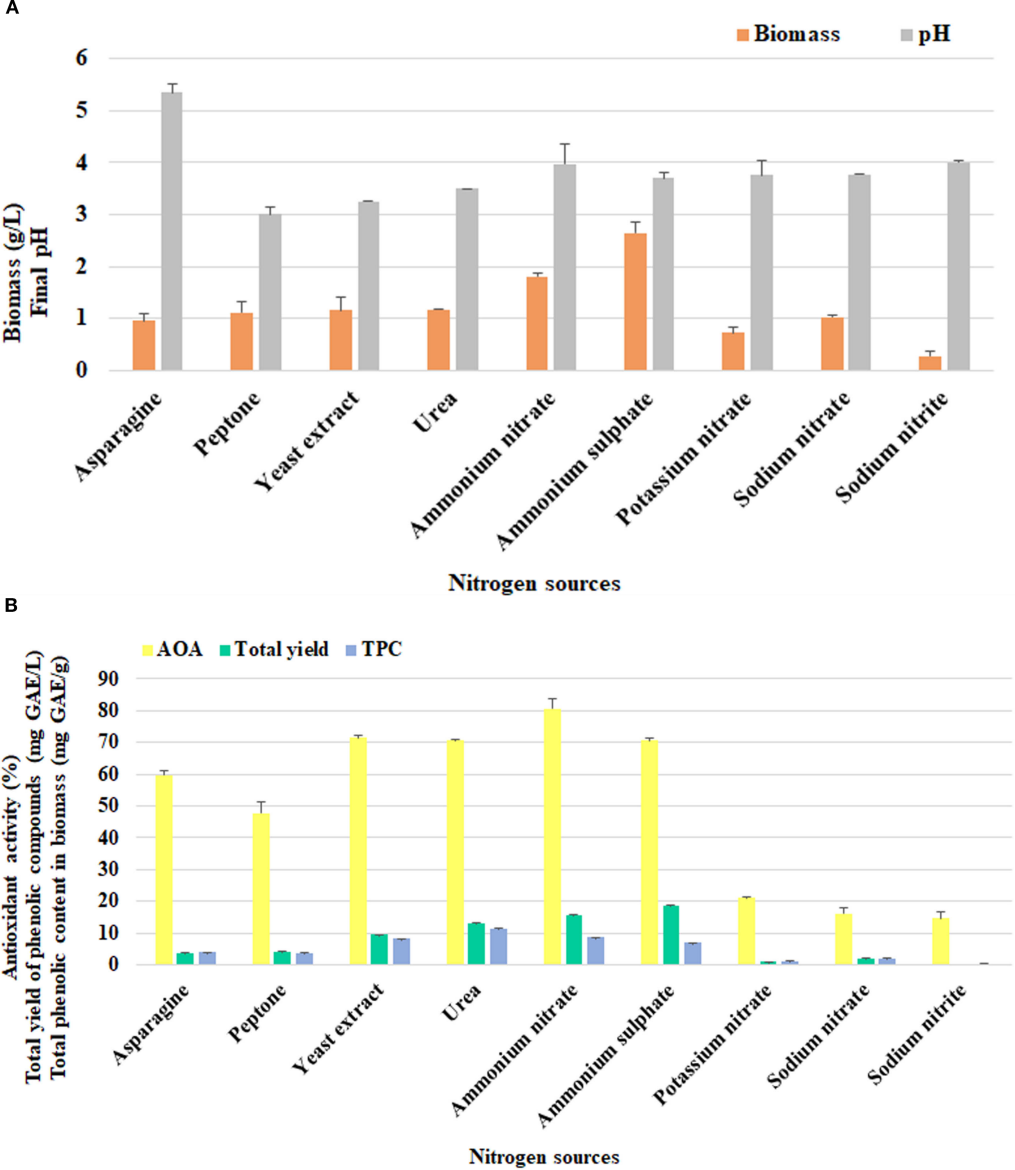
The effect of nitrogen nutritional sources on *F*. *betulina*: **(A)** biomass growth and final pH of cultural media; **(B)** phenols accumulation level and AOA.

A total of nine organic and inorganic nitrogen compounds were screened for *F. betulina* cultivation. Different preferences for nitrogen sources were observed in the growth of *F. betulina* (**Figure 5A**). Ammonium salts were found to be the most effective in supporting biomass formation, with the highest biomass production achieved using ammonium sulfate (2.64±0.21 g/L). The fungal growth did not differ significantly when organic nitrogen sources were used, compared to the application of sodium nitrate. The least effective nitrogen source for biomass production in this study was sodium nitrite.

As a result of incubation, the culture liquid generally became strongly acidic, with pH values ranging from 3.0 to 4.0. The only exception was observed in the asparagine medium, where the pH remained higher at 5.4, indicating a less acidic pH under these specific conditions.

Notably, the results for AOA and TPC levels were partially consistent with mycelial growth results (**Figure 5A**), in contrast to the findings for carbon nutritional sources. Ammonium nitrate helped to express the highest AOA (80.54±3.10%) (**Figure 5B**). However, ammonium sulfate, urea and yeast extract also supported significant free radical scavenging by *F. betulina* extracts (≥70%). At the same time, urea promoted the highest phenolic concentration in biomass (11.32±0.05 mg GAE/g). The highest phenolic yield, normalized to culture volume, was achieved with ammonium sulfate (18.43±0.18 mg GAE/L).

### 3.6 Optimal initial pH level

**Figure 6:**
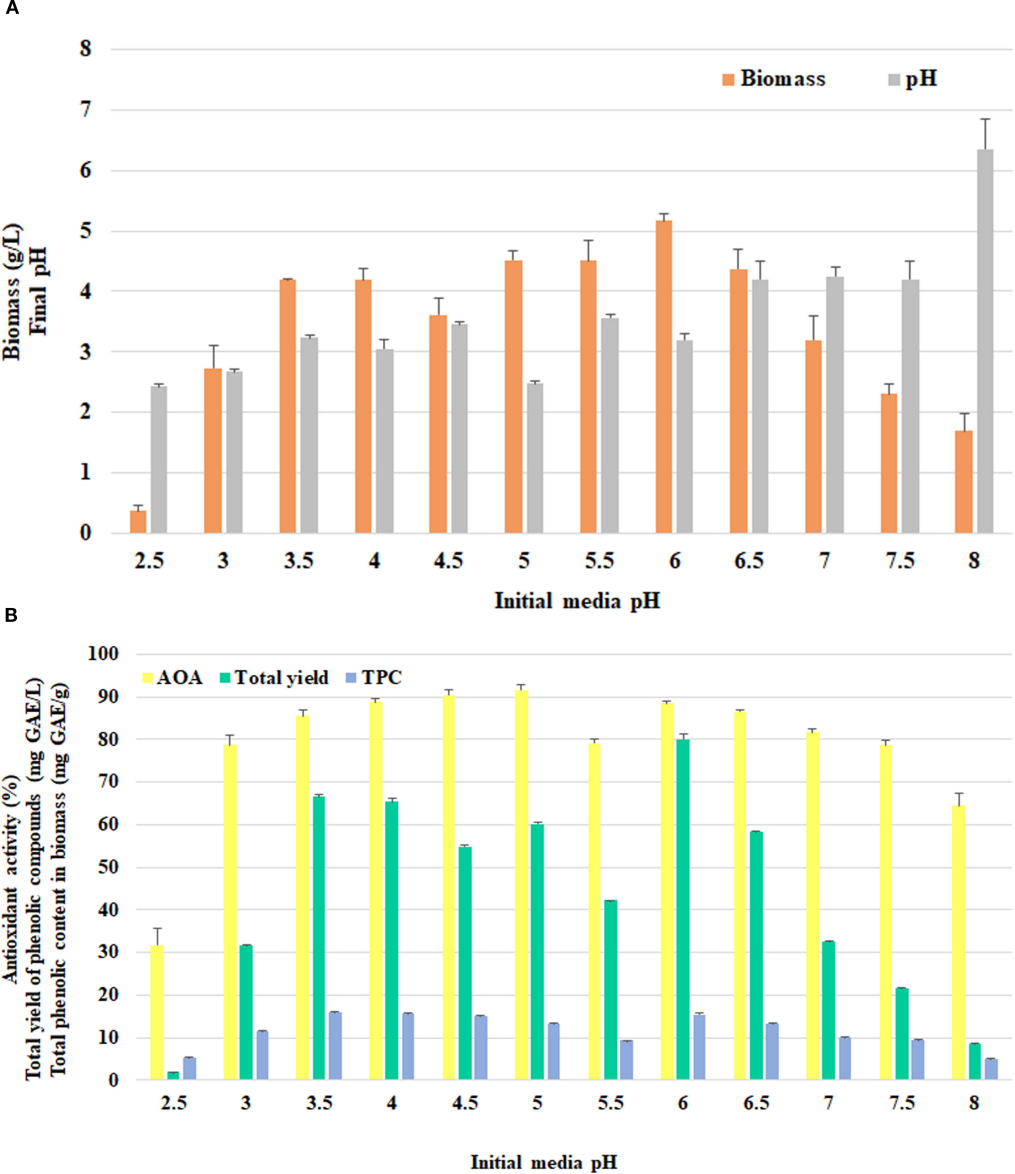
The effect of initial media pH on *F*. *betulina*: **(A)** biomass growth and final pH of cultural media; **(B)** phenols accumulation level and AOA.

*F. betulina* was able to grow at a wide range of pH values, from 2.5 to 8.0 (**Figure 6A**), with the largest amount of mycelium in the range from 5 to 6.5. Following the cultivation, the final pH for the most acidic media (pH 2.5–3.5) practically did not change. A regular drop in the moderate acidic media (pH = 4.0–6.0) was observed with moderately to slightly acidic initial media where the pH dropped to 2.5–3.5. Neutral (6.5–7.5) media resulted in a final pH value of 4.2–4.3. Аlkaline medium (initial pH = 8.0) was the most difficult for the isolate to neutralize, reaching pH 6.4 at the end of cultivation.

*F. betulina* exhibited the best biomass growth at a pH of 6.0 (5.17±0.11 g/L) (**Figure 6A**). AOA of 85% was achieved within the initial pH range of 3.5–5.0 and 6.0–6.5, with the best result occurring at pH 5.0 (91.60±1.15%) (**Figure 6B**). The maximum phenolic concentration was detected in the medium by the mycelium after growth at pH 3.5, while the largest total phenolic were harvested at pH 6.0.

### 3.7 Correlation between the levels of biomass, TPC, and AOA values under different cultivation conditions

The Pearson coefficient (r) was applied to establish potential interrelations between fungal growth and the assessed parameters (**Table 2**). Accordingly, both positive and negative correlations of varying strength were identified.

**Table 2:** Pearson correlation coefficient (r) between growth and the assesed parameters of *F. betulina* depending on cultivation conditions.

Correlation	Extractants	Temperature	Cultivation		Nutritional sources		pH
			Static	Dynamic	Carbon	Nitrogen	
Biomass/AOA	–	-0.8036	-0.4427	-0.06738	-0.157	0.07901	-0.6504
Biomass/TPC	–	0.5994	0.7172	0.7395	0.3455	0.3238	0.3637
AOA/TPC	-0.08253	-0.8858	-0.1457	-0.1315	-0.08271	0.02617	-0.1694

Description of correlation level: |r|<0.1 – very small, 0.1≤|r|<0.3 – small, 0.3≤|r|< 0.5 – medium, 0.5≤|r| – large (according to Cohen, 1988).

The strongest positive correlations were found between biomass accumulation and TPC under changes of temperature (r = 0.5994) and cultivation time (both static and dynamic, with r = 0.7172 and r = 0.7395, respectively). These parameters also were positively related when different carbon and nitrogen sources were used, as well as changing level of pH, but moderately (r = 0.3455, r = 0.3238, and r = 3637, for each parameter, respectively).

However, inverse relationships also occurred in the assessed parameters. A large negative correlation was detected between AOA and biomass (r = -0.8036), as well as between AOA and TPC (r = -0.8858), in response to temperature impact. Additionally, strong (r = -0.6504) and medium (r = -0.4427) negative interrelations were found between AOA and biomass under different pH levels and durations of static cultivation, respectively.

## 4 Discussion

*F. betulina* (commonly known as the Birch polypore fungus) has attracted the attention of many mycologists, biotechnologists, and pharmacologists due to its known biological activity supported by centuries of use in traditional medicine and modern studies that have often focused on antibacterial, antitumor, anti-inflammatory, and immunomodulatory activities. Overall, studies on *F. betulina* cultivated *in vitro* remain limited compared with the extensive research on wild-grown fruiting bodies. Nevertheless, a significant portion of these studies were devoted to the impact of cultivation conditions on the mycelial growth of this species (Lomberh et al., 2002; Mykhaylova, 2014; Dresch et al., 2015; Krupodorova et al., 2016, 2018, 2019; Mykchaylova et al., 2021). Also, there is a lack of knowledge regarding the antioxidant activity of *F. betulina*, becoming particularly popular in recent decades due to the growing interest in healthy lifestyles, disease prevention, anti-aging, and the search for natural antioxidants that can help address these issues (Itrat et al., 2025). Our previous study evaluated the strain-specific properties of 22 isolates of *F. betulina* and identified the strain 2777 as the most promising for antioxidant activity and phenolic content (Zaichenko et al., 2025).

Micro- and macromorphological investigation of mycelium are a fundamental requirement for biotechnological used of strains. It is also important to monitor the purity of the culture, as well as to observe its phenotype. The established microstructures of the *F. betulina* mycelium are representative of this species and have been previously described in early studies (Buchalo et al., 2009; Mykhaylova, 2014; Badalyan and Gharibyan, 2017; Mykhaylova et al., 2021). Generally, *F. betulina* cultures form morphologically distinct colonies due to strain specificity and the cultivation medium (Mykhaylova, 2014; Badalyan and Gharibyan, 2017; Mykhaylova et al., 2021). However, colony morphologies of some *F. betulina* strains similar to our results are reported by Mykhaylova (2014).

One of important factor to consider in order to increase the yield of the desired metabolite is the choice of solvent. The selection of the optimal solvent for extracting the target biologically active substances was not conducted with a specific, intentional focus. A range of extraction solvents have been utilized to isolate bioactive compounds from various *F. betulina* materials (fruiting bodies, submerged biomass, and culture liquids), as reported in the literature. Previous studies on fruiting bodies have employed polar solvents such as water (Keller et al., 2002; Vunduk et al., 2015; Pleszczyńska et al., 2016, 2017; Alresly, 2019; Szymański et al., 2019), alkali (Vunduk et al., 2015), acids (Sułkowska-Ziaja et al., 2012, 2018), ethanol (Cyranka et al., 2011; Dresch et al., 2015; Pleszczyńska et al., 2016, 2017), and methanol (Keller et al., 2002; Reis et al., 2011; Pleszczyńska et al.,2017; Sułkowska-Ziaja et al., 2012, 2018; Alresly, 2019; Kozarski et al., 2024), as well as non-polar options including ether (Cyranka et al., 2011; Pleszczyńska et al.,2017), ethyl acetate (Alresly et al., 2016), hexane (Alresly, 2019), dichloromethane (Keller et al., 2002; Sułkowska-Ziaja et al., 2018), chloroform (Pleszczyńska et al., 2017; Sułkowska-Ziaja et al., 2018), and benzene (Pleszczyńska et al., 2017). Similar solvent choices have also been applied to mycelia (Sułkowska-Ziaja et al., 2018; Alresly, 2019) and culture liquids (Schlegel et al., 2000; Alresly, 2019), demonstrating a diverse methodological landscape for compound extraction.

Selecting the right solvent can significantly enhance the extraction process, maximizing the quantity of the desired compounds. It was found that endopolysaccharides, which exhibit antioxidant activity (Zhao et al., 2023), can be both precipitated by ethanol and fully extracted by it (Abd El-Zaher et al., 2015). Some fungal polysaccharides are water-soluble (Kumla et al., 2025), which could explain the difference between high AOA and low TPC in the water extract. The diversity of phenolic compounds and the differences in their proportions in fungal extracts must also be considered, as these factors may affect AOA levels (Sułkowska-Ziaja et al., 2012, 2018; Kozarski et al., 2024). Tocopherols, carotenoids and ascorbic acid can be produced by Birch polypore and have antioxidant potential (Reis et al., 2011; Kozarski et al., 2024). The lack of correlation between AOA and TPC in *F. betulina* can be attributed to the non-specific nature of the Folin – Ciocalteu assay, which may also detect non-phenolic antioxidants, as well as qualitative differences in the phenolic composition of the sample. This finding is not consistent with the results reported in other similar correlation calculations (Szymański et al., 2019; Kozarski et al., 2024; Krupodorova et al., 2024a) and direct studies of the biological role of *F. betulina* phenolic compounds (Li et al., 2024).

In order to make an informed choice of solvents for the extraction step of *F. betulina* mycelium, their polarity, toxicity and specificity of the extraction process requirements were taken into account. High antiradical activity and phenols content were found when using 70% ethanol, methanol and water. Based on the results of the screening, the optimal polarity of 70% ethanol and methanol provided the best conditions for the extraction of phenols due to its ability to dissolve both polar and non-polar molecules. At the same time, 96% ethanol may be less effective in extraction than 70% due to its strong dehydrating capacity that can coagulate mycelial proteins, which prevents the solvent for penetrating the fungal cell. In contrast, water-containing ethanol causes slower coagulation of cell walls and improves solubility. In addition, water, although capable of extracting polar phenolic compounds, does not dissolve non-polar or more complex molecules effectively. The conducted studies confirmed that methanol was the most effective solvent for obtaining *F. betulina* extracts with high free radical inhibition capacity and phenolic content, outperforming 96% ethanol in these aspects. During solvent screening, it was found that 70% ethanol also extracted a relatively significant amount of phenols. This solvent may be able to extract different compouds from mycelium obtained under different cultivation conditions, which could explain the convergence of high levels of AOA and TPC, as well as their disproportion under certain *F. betulina* strain 2777 cultivation conditions. For instance, a study with the mycelial extracts of *Xylaria polymorpha* and *X. longipes* strains extracted with ethyl acetate, 96% ethanol, and methanol, showed the latter exhibited the highest phenolic content (Atamanchuk and Bisko, 2023). In terms of antiradical activity, methanol was the most effective solvent for *X. longipes* strains, while ethanol was more suitable for *X. polymorpha* strains. However, given to the important aspect such as toxicity of the solvents such as methanol and a small difference in antiradical activity, in our study, 70% ethanol was chosen for the subsequent stages of the study focused on cultivated conditions.

During growth, fungi produce a variety of metabolites, the amount of which varies significantly depends on the cultivation conditions, studying these conditions *in vitro* to optimize the process of obtaining bioactive compounds is an important step of cultivation. To achieve this, a single-factor experimental design was used, where each cultivation condition (temperature, pH, carbon and nitrogen sources, static or shaking) was varied one at a time while keeping others constant. This approach was applied for the first time in the case of *F. betulina*, and the study confirmed that it is possible to optimize both its growth and phenolic production, as well as enhance its antiradical activity under controlled conditions.

With regards to the level of biomass and phenols production as well as antiradical activity of *F. betulina*, the optimal cultivation conditions were established as 25 °C for 14 days under static cultivation in medium with xylose and ammonium sulfate at 6.0 pH. The same optimal cultivation temperature was favorable for the growth of another *F. betulina* strains (Dresch et al., 2015). Notably, this temperature is also consistent with the strain-specific characteristics of *F. betulina* and the established optimal growth ranges from 25 to 28 °C (Mykhaylova, 2014). A narrow temperature range (26–28 °C) was also found to be favorable for biomass production and other biological activity (antibacterial) of the *F. betulina* 327 strain during liquid cultivation (Krupodorova et al., 2019).

*In vitro* fungal biomass is mostly obtained using liquid submerged cultivation (Elisashvili, 2012; Bakratsas et al., 2021; Romero et al., 2025). This method has been used to produce antibiotics such as piptamine (Schlegel et al., 2000) and other biologically active agents (Suay et al., 2000; Sułkowska-Ziaja et al., 2018; Alresly, 2019). However, static incubation in liquid media is also used for macromycetes, although it is less common compared to other methods (Suparmin et al., 2019), and there are also similar cases involving Birch polypore fungus (Krupodorova et al., 2016, 2018, 2019; Mykchaylova et al., 2021; Zaichenko et al., 2025). Static cultivation was more effective compared to shaking conditions for obtaining extracts with higher antioxidant activity and TPC formation of *Xylaria feejeensis* (Rebbapragada and Rajagopal, 2016), in contrast to similar studies involving *Fomitopsis pinicola* (Krupodorova et al., 2024a). The difference in growth during submerged cultivation under shaking and static conditions is related to the availability of oxygen, nutrient distribution, and the physical stress applied to the mycelium in each condition. It is important that the selection of the cultivation method for fungi should be considered during the optimization process due to its potential impact on metabolic pathways. It was shown that the differences in the intensity of various metabolic pathways, such as oxidative phosphorylation, the pentose phosphate pathway, heme biosynthesis, and ergosterol biosynthesis, based on the location of *Cordyceps militaris* mycelium (aerial vs. submerged), reflect the fungus adaptive strategies to different conditions, potentially linked to the upregulation or downregulation of relevant pathways (Suparmin et al., 2019).

It is noteworthy that the *F. betulina* strain in this work was characterized by a rapid achievement of a plateau in the dynamics of fungal growth under static and shaking conditions on days 14 and 11, respectively (**Figure 5**). This finding indicate that the fungus is making the most efficient use of available medium nutrients. This may also point the stability and maturity of the cultural conditions for its cultivation. From an economic perspective, the rapid stabilization of *F. betulina* growth at a plateau enables yield prediction, which is advantageous for process optimization and scale-up. The optimal incubation period for fungi depends on the growth and metabolism of the individual culture and may vary due strain-specific characteristics of the fungi. When comparing the static cultivation of *F. betulina* 327 (Krupodorova et al., 2019) and the studied strain, an optimal incubation period of 14 days was observed for both. However, the *F. betulina* 327 accumulates more biomass (8.1±0.1 g/L) than *F. betulina* 2777 (5.28±0.15 g/L). Results of TPC and percentages of antiradical activity of the extracts from *F. betulina* mycelium were comparable with those previously found in similar study of *F. pinicola* (Krupodorova et al., 2024a) under dynamic shaking conditions. However, a shorter cultivation period (14 days) for *F. betulina* was more suitable for total phenolic content (TPC) and antiradical activity, in contrast to *F. pinicola*, which required 28 days in static conditions. A high percentage of antiradical activity of *F. betulina* was also observed on day 35 of static incubation. Therefore, secondary and tertiary metabolites secreted by Birch polypore fungus during prolonged cultivation also play a role in its biological activity, particularly in the mycelium. This is also in line with another study focused on the antibacterial potential of another strain of *F. betulina* 327 (Krupodorova et al., 2019).

Strain specificity of *F. betulina* in consuming different carbon sources has been previously established (Kizitska et al., 2024). In current study, it was found that maltose positively affects the biomass growth of *F. betulina* 2777, which is consistent with our previous results of the growth screening of 22 cultures, which showed better growth of most strains on malt extract medium. Different strains of *F. betulina* share the ability to grow on all the carbon sources used, as well as an expected preference for certain sources (Krupodorova et al., 2019; Mykchaylova et al., 2021), which may be genetically determined. Xylose promoted the mycelium growth of certain macromycetes species such as *Antrodia cinnamomea*, *Calocybe indica*, *Ganoderma lucidum*, *Grifola frondosa*, *Hericium erinaceus*, *Inonotus obliquus*, *Oudemansiella radicata*, *Phellinus torulosus*, *Pleurotus ostreatus*, *Schizophyllum commune*, and *Tricholoma terreum* (Krupodorova et al., 2021). Despite of mediocre biomass (0.99±0.21 g/L) accumulation of *F. betulina* 2777 on xylose, antiradical activity of this strain was the best after growth on this carbon source. A similar trend was observed in our previously study of *F. pinicola* 1523 (Krupodorova et al., 2024a). The results of antiradical activity and TPC in the 96% ethanol extract of *F. pinicola* 1523 mycelium obtained by growth on xylose medium are comparable to the data obtained in the case of the 70% ethanol extract of *F. betulina* 2777. The optimal carbon sources for biomass accumulation and the biological activity of the studied strain differ. This may be due to differences in the conversion of hexoses and pentoses (such as xylose), as well as the lower nutritional value of certain compounds individually for this culture. This can cause stress in the fungus and activate protective biological mechanisms. In contract to our results, glucose was suitable for phenols synthesis and AOA for *Leucopaxillus giganteus* (Barros et al., 2008) while dextrose was the best source for AOA of *Xylaria feejeensis* (Rebbapragada and Rajagopal, 2016).

The effect of nitrogen sources in nutritional media is also meaningful. The *F. betulina* strain 2777 showed specificity in the consumption of ammonium salts (i.e. ammonium sulfate and nitrate), in terms of both growth, TPC and AOA. According to previous studies, this trend is not typical for Birch polypore. A study with 11 *F. betulina* strains showed that the preferred nitrogen source was peptone, and 8 of them also converted asparagine, and only one culture accumulated biomass on ammonium salt such as ammonium hydrophosphate (Mykchaylova et al., 2021). Asparagine was the most effective nitrogen source for biomass accumulation of *F. betulina* 327, although the ammonium sulfate medium promoted growth of fungus (Krupodorova et al., 2019). These results emphasize the importance of strain specificity in the growth of the Birch polypore fungus. Similar to *F. betulina* 2777, certain fungal species also tend to consume ammonium salts such as ammonium acetate for *Agaricus bisporus*, *G. lucidum*, *H. erinaceus*, *Phellinus tremulae*, *Pleurotus eryngii*; ammonium sulfate – for *Ganoderma applanatum*, *G. lucidum*, *Inonotus obliquus*, *Macrolepiota mastoidea*; ammonium phosphate – for *G. lucidum*, *Phellinus alni*, *P. baumi*, *P. cavicola*, *P. chrysoloma*, *P. conchatus*, *P. lundellii*, *P. pomaceus*, *P. populicola*, *P. torulosus*, *P. vorax*; diammonium phosphate – for *Russula sanguinaria*, *Suillus collinitus*, *S. granulatus*, *Tricholoma batschii*, *T. imbricatum*; and ammonium chloride – for *Lentinula edodes*, *Pleurotus cystidiosus*, *P. floridanus*, *P. ostreatus*, *P. sajor-caju* (Krupodorova et al., 2021).

Additionally, *F. betulina* 2777 showed greater versatility in the consumption of nitrogen sources to achieve high levels of AOA and TPC than in the case of carbon sources. Besides ammonium salts, yeast extract and urea also supported the assessed parameters of mycelium. It should be emphasized that organic components (yeast extract, urea, asparagine, peptone) are also sources of carbon nutrition, and ammonium sulfate also contains sulfur (which may also be present in proteins with sulfur-containing amino acids in yeast extract and peptone). Therefore, further selection of the C:N ratio in the nutrient medium, as well as the use of sources of other macro- and microelements, is relevant for macromycetes. In contract, peptone improved for AOA and TPC of *F. pinicola* (Krupodorova et al., 2024a), whereas yeast extract was raised for the free radical scavenging activity and TPC of *Xylaria feejeensis* (Rebbapragada and Rajagopal, 2016). It should be noted that nitrogen sources affect not only the free radical scavenging ability of Birch polypore, but also its antibacterial activity (Krupodorova et al., 2019). Growth inhibition studies of *Bacillus subtilis*, *Escherichia coli* and *Staphylococcus aureus* under the influence of the culture liquid and inhibition of *B. subtilis* and *E. coli* by mycelium of *F. betulina* 327 was established in the presence of ammonium nitrate, when using ammonium sulfate, the antibacterial activity was absent. In contrast, corn extract and peptone were the best nitrogen sources for the growth of *Pleurotus ostreatus* PBS281009 and *Coprinus comatus* M8102 as well as for scavenging ability of their ethanolic extract from lyophilized mycelium (Vamanu, 2013).

The pH level is also crucial parameter for cultivation due to its impact on fungal metabolism and growth. This study demonstrated the ability of *F. betulina* to grow at a wide range of pH levels (from 2.5 to 8.0). Biomass production was comparable at pH 5.0–5.5 (4.5 g/L) to another study (Mykchaylova et al., 2021), whereas at pH 6.0 it exhibited higher level (5.17±0.11 g/L). This may occur due to strain-specific factors and variations in nutrient medium composition across different studies. Nevertheless, the *F. betulina* 327 accumulated high levels of biomass (>10 g/L) at pH 3.5–4.0 in the same liquid glucose peptone yeast medium (Krupodorova et al., 2019); lower pH values were suboptimal. Lomberh et al. (2002) studied a narrower range of acidity levels (3.2–6.1) for the *F. betulina* 1653 strain. At pH 3.2, the culture produced >9 g/L of biomass; however, as the pH level increased, the strain’s growth was gradually inhibited.

The biological activity of some cultures is less affected by changes in pH. The investigated strain exhibited high (>75%) antioxidant activity over a wide pH range (3.0–7.5). This finding positions the strain as versatile and resistant to various stress factors, which significantly expands its potential in biotechnology, medicine, and industry. *F. pinicola* 1523 exhibited an AOA level of over 80% in the pH range of 2.5–6.0, with the maximum formation of phenolic compounds (>25 mg GAE/g) occurring at pH levels of 2.5–5.0 (Krupodorova et al., 2024a). In contrast, the studied *F. betulina* strain was susceptible to pH levels affecting phenolic formation. TPC values ≥15 mg GAE/g were observed at pH levels of 3.5–4.5 and 6.0. Also, the *F. betulina* 327 strain exhibited antibacterial activity in the culture liquid at all the pH values studied (3.5–6.5), although it still preferred slightly acidic conditions, which increased its ability to inhibit the test microorganisms at pH 5.5–6.5 (Krupodorova et al., 2019). Therefore, all the results obtained are consistent with the general trend for macromycetes, which mostly prefer slightly acidic or neutral pH values (with rare exceptions) (Krupodorova et al., 2021). However, it is important to adjust the acidity of the nutrient media for each Birch polypore culture individually.

Given that different cultivation conditions significantly affected biomass production, TPC, and AOA of *F. betulina*, changes in temperature and cultivation duration (both static and shaking) had the greatest effect, and these data can be used for further optimization of cultivation. Variation in pH and nutritional sources had less influence. These factors are more stressful and affect the chemical composition of the nutritional medium, which can influence fungal metabolism by directing it towards either biomass synthesis or secondary metabolite production. The obtained results are particularly consistent with the correlation between biomass and TPC in *F. pinicola* (Krupodorova et al., 2024a), in which temperature and the duration of static cultivation were the most significant factors.

In contrast, the amount of biomass correlated negatively with AOA, particularly at different temperatures and acidities. The physical parameters of cultivation (temperature and pH) were likely to stress Birch polypore cultures. This explains why an increase in AOA probably happens simultaneously to low biomass formation, due to increased production of biologically active compounds in order to adapt to the growing conditions. Additionally, the correlation was more affected by cultivation under static conditions than under shaking conditions, perhaps due to their different durations (35 and 15 days, respectively). It should be noted that longer cultivation periods promote secondary metabolism, products of which influence the fungal biological activity (Krupodorova et al., 2019), although they also depletes the nutrient sources. Additionally, during cultivation with shaking, metabolites and dead cells do not accumulate near the fungal mycelium due to more efficient mass transfer, and there is a constant supply of nutrients since the concentrations of substances in the culture liquid are balanced during mixing. In static cultivation, diffusion occurs more slowly, which can create stressful conditions. However, macromycetes are known to grow effectively on solid substrates under static conditions (Berovic et al., 2024), and on liquid ones as well (Rebbapragada and Rajagopal, 2016; Suparmin et al., 2019). Therefore, the effect of each cultivation method should be considered separately.

To calculate the potential correlation between the established values, the Pearson coefficient was applied. The correlations for each *F. betulina* 2777 cultivation parameter can be ranked in terms of influence as follows: temperature > duration of static cultivation > pH > duration of dynamic cultivation > carbon sources > nitrogen sources. These results contrast with those observed for *F. pinicola*, where only positive correlations of varying strength were found (Krupodorova et al., 2024a). Despite this, both species shared similar trends, particularly the significant influence of the duration of static cultivation and the minor role of nitrogen sources. In addition, the possibility of optimizing the growth and phenolic production of *F. betulina*, as well as enhancing antiradical activity under controlled conditions is in line with other studies with such species as *Leucopaxillus giganteus* (Barros et al., 2008), *Coprinus comatus*, *Pleurotus ostreatus* (Vamanu, 2013), *Ganoderma neo-japonicum* (Tan et al., 2015), *Xylaria feejeensis* (Rebbapragada and Rajagopal, 2016). Such research is important as it offers valuable insights into optimizing cultivation conditions, which are key to boosting biomass and bioactive compound production, thereby advancing biotechnological applications.

## 5 Conclusions

The adaptations of fungi are closely related to their physiological and metabolic abilities to different environmental conditions. The current study provides insight into the impact of cultivation on biomass and phenols production, as well as enhancement of free radical scavenging activity of *F. betulina*. Methanol and 70% ethanol proved to be the most effective extraction solvents to phenolics while extracts from all solvents cause strong DPPH inbihition. These results suggest that *F. betulina* could serve as a natural source for the development of dietary supplements aimed at combating oxidative stress. At 25°C, pH 6.0 and static cultivation conditions were suitable for ensuring maximum biomass accumulation, total phenolic yield, and antioxidant activity. Among the carbon sources, maltose contributed to the highest biomass growth, while xylose resulted in the highest levels of TPC and DPPH inhibition. Among the nitrogen sources, ammonium sulfate led to the greatest biomass accumulation, whereas ammonium nitrate enhanced antioxidant activity. Urea provided the highest TPC per gram of dry biomass, while ammonium sulfate yielded the highest total phenolic yield. The established optimal cultivation parameters are key for studying the assessed parameters of *F. betulina*. Further investigation of cultivation optimization using Box-Behnken design, response-surface methodology and artificial neural network-genetic algorithm can give more insights for the biotechnological application of this strain. Limitations of the current study include the lack of complete identification of phenols and detailed metabolite profiling using LC-MS/MS.

## Data Availability

The original contributions presented in the study are included in the article/supplementary material. Further inquiries can be directed to the corresponding author.
